# Dynamics of Insects and Their Facultative Defensive Endosymbiotic Bacteria: A Simulation Model

**DOI:** 10.1002/ece3.70676

**Published:** 2024-12-03

**Authors:** Eric Wajnberg, Fernando L. Cônsoli

**Affiliations:** ^1^ INRAE Sophia Antipolis Cedex France; ^2^ INRIA Projet Hephaistos Sophia Antipolis Cedex France; ^3^ Departamento de Entomologia e Acarologia USP/ESALQ Piracicaba Brazil

**Keywords:** efficacy of biological control, host aggregation, insect symbionts, Monte Carlo simulations, wasp females' longevity

## Abstract

Most insects harbour endosymbionts that modify their physiology, reproductive mode, and ecology. One fascinating case is in aphids, which host endosymbionts that protect them against attacks from parasitoids. These symbionts are transmitted maternally with high fidelity but can also be transmitted horizontally from infected to uninfected hosts. Since symbionts can confer resistance to their host against parasitoids, levels of symbiont infection should rapidly spread to fixation. This is not the case in most aphid populations that have been studied. Furthermore, the defensive effect of symbionts has been thought to reduce the efficacy of biological control against crop pests, although this has never been properly quantified. We developed a Monte Carlo simulation model to examine changes in levels of endosymbiont infection in an insect population in the presence of parasitoids attacking them over several generations. We also used the model to quantify potential reductions in the efficacy of parasitoids in controlling host populations in biological control. Results suggest that longevity of parasitoids and the spatial aggregation of hosts likely play a major role in the dynamics of symbiont infection. This is the first evidence that these ecological parameters are potentially important for explaining levels of symbiont infection in insect populations.

## Graphical abstract

Aphids host endosymbionts protect them against attacks from parasitoids, leading to potential reduction in the efficacy of biological control against these crop pests. We developed a Monte Carlo simulation model to examine changes in levels of endosymbiont infection in an insect population in the presence of parasitoids attacking them over several generations, and to quantify potential reductions in the efficacy of parasitoids in controlling host populations in biological control. Results suggest that longevity of parasitoids and the spatial aggregation of hosts likely play a major role in the dynamics of symbiont infection.

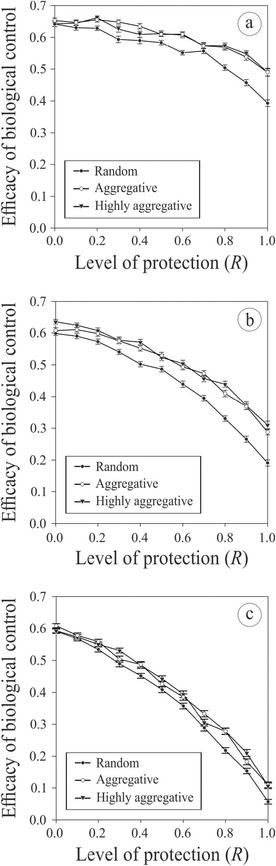

## 1 Introduction

The life histories of most insects depend heavily on the intracellular bacterial endosymbionts they host (Douglas 1989; Moran, McCutcheon, and Nakabachi 2008; Feldhaar and Gross 2009). Such insect–endosymbiont relationships have a long evolutionary history that has likely spanned several million years (Moran et al. 1993; Cornwallis et al. 2023), leading to intimate associations in which insects and their symbionts have developed cooperative interactions (Douglas 2015). Endosymbionts can either be obligatory (necessary for the survival and reproduction of their hosts) or facultative (non‐essential) (Moran, McCutcheon, and Nakabachi 2008). Over the past decades, there is growing evidence that symbionts can, among other roles: (1) provide nutritional compounds (e.g., Ankrah, Luan, and Douglas 2017), (2) improve detoxification, immunity and digestion (e.g., van den Bosch and Welte 2017; Brown et al. 2020) or (3) modify the reproductive mode and/or be required for oogenesis (e.g., Dedeine et al. 2001; Werren, Baldo, and Clark 2008) of their hosts. More generally, they can have a major influence on the ecology and evolution of their hosts, with cascading effects on entire populations and species communities (Moran 2008; Oliver et al. 2010; Ferrari and Vavre 2011; Brown et al. 2020; Frago and Zytynska 2023). Hence, studying both the distribution of symbionts within and among the species that host them, and their functions, can contribute to a better understanding of their communities and, for example, of how they are involved in the interactions between insects and their pathogens (Vorburger and Perlman 2018; Brown et al. 2020).

One of the most fascinating effects of endosymbionts on their hosts is that they can confer, to a certain extent, resistance to pathogens (Scarborough, Ferrari, and Godfray 2005; Rothacher, Ferrer‐Suay, and Vorburger 2016; Gurung, Wertheim, and Falcao Salles 2019). In this respect, aphids are an excellent model system for studying insect–symbiont interactions (Flórez et al. 2015). Like many insects feeding on plants, aphids can be infected with facultative endosymbionts that are known to affect their reproductive efficiency, and especially their resistance to insect parasitoids (Oliver, Smith, and Russell 2014; Vorburger 2014; Zytynska, Thighiouart, and Frago 2019). The most studied example is the association between the pea aphid *Acyrthosiphon pisum* (Hemiptera: Aphididae), and one of its most common facultative symbionts, the γ‐proteobacteria *Hamiltonella defensa* (Oliver et al. 2003, 2010; Vorburger 2022). The protection provided by the endobacteria against attacks from the parasitoid *Aphidius ervi* (Hymenoptera: Braconidae) is due to a Podoviridae bacteriophage encoding a toxin protein that kills the developing parasitoid larvae within its hosts (Degnan and Moran 2008; Oliver et al. 2009). In this system, the level of protection provided by the endosymbionts have been shown to be highly variable (from 0% to 100%), depending on the genotypes of the aphid, the bacteriophage and the parasitic wasp (Oliver, Moran, and Hunter 2006; Oliver et al. 2010; Oliver and Higashi 2019).

Symbionts are mainly transferred vertically to the aphid progeny through transovarial transmission with nearly 100% efficacy, but they can also be transferred horizontally, both within and among species (Henry et al. 2013; Oliver and Higashi 2019; Kaech and Vorburger 2021). The mechanisms of symbiont horizontal transmission between hosts are not always accurately known (Brown et al. 2020). Possible routes have been shown to be: (1) during sexual reproduction (Moran and Dunbar 2006); (2) through parasitoid females attacking infected and uninfected hosts (Brown et al. 2020) or (3) via feeding on a common diet, through plant tissues and surface contamination (Darby and Douglas 2003). Although rates of horizontal transmission are most likely difficult to be estimated under natural conditions (Kwiatkowski and Vorburger 2012), they are reportedly highly variable (Moran and Dunbar 2006; Kwiatkowski and Vorburger 2012). Horizontal transmission probably requires the donor and the receiver to be in close proximity, although this has not yet been formally demonstrated (Huigens et al. 2004; Palma et al. 2022).

Hosting symbionts provides an obvious benefit for hosts if they confer resistance to parasitoid attacks. However, because levels of symbiont infection usually remain at intermediate values in most aphid populations, it is most likely that hosting defensive endosymbionts also bears some costs, as shown in population cage experiments (Oliver et al. 2008; Dykstra et al. 2014). The estimated magnitude of these costs ranges between about 10% and 40% of aphids' lifetime reproduction (Vorburger and Gouskov 2011; Kwiatkowski and Vorburger 2012).

Insect parasitoids have been used in biological control programmes for many years, often successfully, to control aphids on different crops, especially in greenhouses (Boivin, Hance, and Brodeur 2012). However, because hosting endosymbionts can confer resistance to aphids against parasitoids, releasing insect parasitoids may select for an increase in host infection levels (Sanders et al. 2016), eventually compromising the efficacy of biological control programmes (Oliver et al. 2010; Käch et al. 2018; Vorburger 2018; Rossbacher and Vorburger 2020). This phenomenon has actually never been demonstrated under field conditions, but it has nonetheless triggered concerns and debates over the last decade, and several strategies have been proposed to circumvent it. For example, there have been proposals to combine the release of parasitoids with other biological control agents (Vorburger 2018), and/or to increase plant biodiversity in the surrounding landscape (Zytynska and Meyer 2019).

From a population and evolutionary dynamics point of view, interactions between insects and their endosymbionts are still not yet fully understood. Several puzzling questions remain unanswered (Schmid‐Hempel 2003). For example, several authors have tried to find theoretical explanations about why intermediate infection levels are observed (Kwiatkowski and Vorburger 2012; Foxall 2019; Preedy et al. 2020; Palma et al. 2022). In this respect, none of the past studies explicitly considered the spatial structure of the interactions between host insects and their symbionts. This is despite the fact that, as mentioned above, horizontal transmission most likely works only if hosts are in close proximity. Furthermore, these theoretical approaches also did not consider effects on the efficacy of biological control programmes using insect parasitoids resulting from these endosymbiont infection dynamics.

We developed an individual‐based Monte Carlo simulation model that explicitly considers the spatial distribution of infected and uninfected hosts, taking into account the main ingredients of the interaction between hosts and their endosymbionts (vertical and horizontal transmissions, level of protection conferred by the symbionts, cost of being infected, etc.). The goal was to simulate the dynamics of infection during several host generations in the presence of parasitoid females. Although the model presented here implicitly refers to the association between the parasitoid *A*. *ervi*, its host *A. pisum* and the endosymbiont *H*. *defensa* (and the associated bacteriophage), it is not explicitly parameterised for this host‐endosymbiont‐parasitoid system, and can then be generalised to any kind of host‐endosymbionts interaction sharing the same ecological characteristics. The results demonstrate that taking into account explicitly the spatial distribution of the hosts (especially their proximity) can influence the dynamics of the infection. We also demonstrated that the longevity of parasitoid females likely plays a crucial role in determining levels of symbiont infection. Finally, the consequences in terms of the efficacy of biological control programmes are discussed.

## 2 Description of the Model

Several modelling frameworks can be used to study parasitoid‐host‐symbiont interactions, and all the theoretical models developed so far (Kwiatkowski and Vorburger 2012; Foxall 2019; Preedy et al. 2020; Palma et al. 2022) have been based on differential equations. However, since the goal of the model presented here is to explicitly account for the proximity between individuals to model symbiont horizontal transmission, we opted for an individual‐based Monte Carlo simulation model (Wajnberg 2023). The model is used to study the evolution of the endosymbiont infection rate in a population of hosts along several generations, with the presence of parasitoid females attacking them.

Hosts, infected or uninfected, and their parasitoids are located in a 2D square grid of 500 × 500 cells. 200 hosts are used in all simulations, and this number remained fixed for all generations. Hosts' location at the beginning of each generation is drawn randomly in the grid, subject to the constraint that each grid cell can contain at most one host. A proportion p of the hosts are infected by endosymbionts, and this proportion is fixed at the beginning of each simulation. The identity of the hosts that are infected (if any) is drawn randomly to obtain an aggregated spatial distribution in patches. For this, a two‐step process is used: the number of patches of infected hosts corresponds to a given percentage a of these infected hosts and each patch contains the same number of infected hosts. The centre of each patch is drawn randomly in the grid defining the environment. Hence, using different values of a can lead to different levels of infected host aggregation, from all infected hosts located in one patch only (which would be the case, for example, if there are 200 infected hosts with a=0.5%), to each infected host belonging to a different patch (random spatial distribution; which would be the case, for example, if there are 200 infected hosts with a=100%). Then, the hosts that are closest to the patch centres are considered to be infected. Since we wanted to test the overall effects of the aggregation level of infected hosts, such an algorithmic procedure is used in all generations, leading to a constant aggregation level in each simulation. Hosts—here, considered to be aphids—are fixed in each generation and are thus not moving during the simulation, which approximately corresponds to what is observed on real insects. The longevity and fecundity of all uninfected hosts are drawn randomly at each generation from Exponential and Poisson distributions, with averages $S_{\mathrm{un}}$ and $\lambda_{\mathrm{un}}$, respectively. When hosts are infected, however, they paid a cost, which is considered to affect both their longevity and fecundity, as this was observed experimentally by Vorburger and Gouskov (2011), and Simon et al. (2011). Costs of being infected can be either constitutive or induced (Kwiatkowski and Vorburger 2012). Constitutive costs are associated to the innate maintenance of the defensive machinery in the host irrespective of whether it is infected or not, while induced costs are paid only when an infection occurs (Kraaijeveld, Ferrari, and Godfray 2002). Here, only constitutive costs are considered. For this, longevity and fecundity of infected hosts are also drawn at each generation from Exponential and Poisson distributions, but with averages $S_{\text{in}}=\left(1-c\right)\times S_{\mathrm{un}}$ and $\lambda_{\text{in}}=\left(1-c\right)\times \lambda_{\mathrm{un}}$, respectively, c (ranging from [0.0; 1.0]) representing the cost of being infected. Hosts that have not been attacked by a parasitoid and that ‘naturally died’ during the simulation then disappear from the grid.

Hosts that are infected (or that became infected through horizontal transmission, see below) all transmit their symbionts to their progeny (100% vertical transmission success rate), which approximately corresponds to what is observed in real situations (Darby and Douglas 2003; Kwiatkowski and Vorburger 2012). Uninfected hosts can become infected through horizontal transmission if they are close enough to an (still alive; see below) infected host. For this, at each time step of the model, the probability of an uninfected host becoming infected follows an exponential decay model: $\exp\left(-\left(1-h\right)\times d\right)$, where d is the distance to each infected host in the vicinity of the uninfected host and h (ranging from $\left[0.0;1.0\right]$) the intensity of horizontal transmission (in arbitrary unit). A host that becomes infected starts to pay an infection cost, with its remaining longevity l and fecundity per time step f replaced by $\left(1-c\right)\times l$ and $\left(1-c\right)\times f$, respectively.

In all simulations and all generations, 10 female parasitoids are always used, and their location is randomly drawn over the grid at the beginning of each generation. Just like for their hosts, the longevity and fecundity of the wasp females are drawn from Exponential and Poisson distributions with averages $S_{p}$ and $\lambda_{p}$, respectively. Females disappear from the simulation at each generation when their lifetime duration ended, or when they laid all their eggs. For the sake of simplicity, and even if this is not the case for *A*. *ervi* (Zhao and Wang 2008), we considered that female parasitoids are proovigenic (i.e., they have all their eggs available at the beginning of their life) and thus do not mature new eggs during their lifetime duration.

During the simulation, parasitoids forage for hosts to attack. Following the results obtained by Oliver et al. (2003) (but see Łukasik et al. 2013) we assumed that parasitoids cannot discriminate between infected and non‐infected hosts and are equally attracted to both of them. At each time step in the simulation, parasitoids are able to perceive the location of all hosts remaining in the grid (i.e., not previously killed by a parasitoid attack) and are attracted to them with a probability that decreases exponentially with their distance. Once a female targets a host, it moves in its direction, each time step at a distance drawn from a Normal distribution with average μ and standard deviation σ, and the process is repeated at each time step. If a female find an unattacked host, it attacks it. If this is an uninfected (symbiont‐free) host, the female lays one egg in it, and the host dies and cannot be perceived by female wasps anymore. In the real world, not all attacks to uninfected hosts are successful, but most of them are, so we think that the process used here correctly represents real situations. If this host is infected, however, it can escape from the parasitoid attack with a probability R that corresponds to the level of resistance conferred by the symbionts. In this case, the parasitoid does not lay an egg in it. Just like in Kwiatkowski and Vorburger (2012), we considered that hosts cannot be superparasitised. If an infected host resists to a parasitoid attack, it can still be perceived again by another parasitoid and still produces aphid progeny.

**FIGURE 1:**
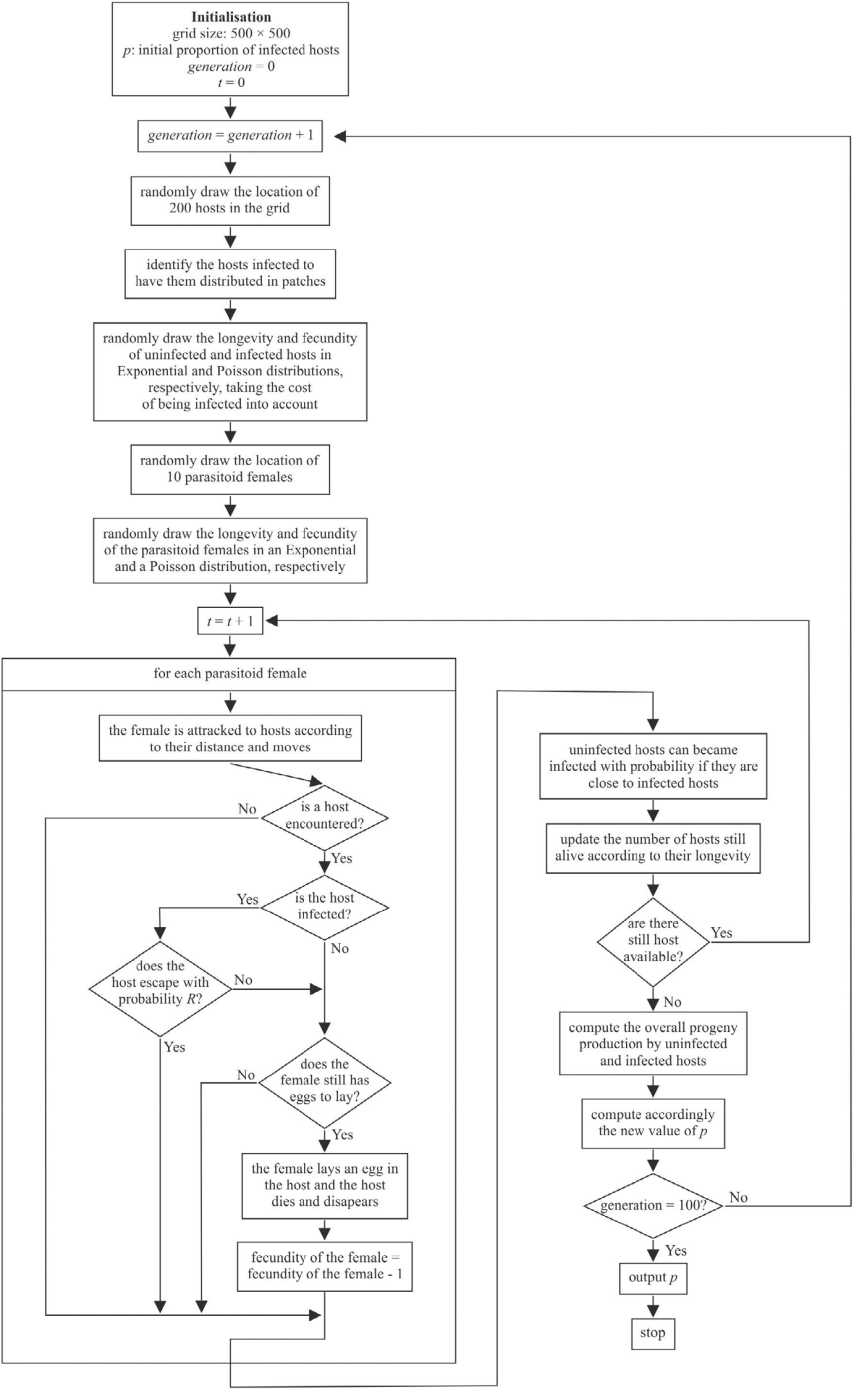
Flowchart of the Monte Carlo simulation model used to understand changes in levels of endosymbiont infection in an insect population in the presence of parasitoids attacking them over several generations.

Each generation stops when all hosts either ‘naturally’ died or were successfully attacked by a parasitoid female. We considered that all hosts in the simulation reproduce asexually, as this is the case in most of the life cycle of aphids (Vilcinskas 2016), and we assumed that they reproduce at a constant rate per time step, which corresponds to the total number of progenies produced by the hosts (infected or not) during their lifetime divided by their overall longevity. Hence, at each time step in the simulation we considered that aphids that are still alive and unattacked produce progeny, enabling us to compute the total number of infected and uninfected progeny at the end of the generation. The frequency of the two types of progeny was then used to set the starting conditions for the next generation, and the simulation is repeated over 100 generations. Several preliminary computations indeed demonstrated that a steady infection level equilibrium was always achieved in 100 generations of simulation. Such a simulation procedure thus enabled the host population to changes in terms of infection rate while the level of resistance to parasitoid attack remained constant along the different generations of each simulation. In other words, in the model, parasitoids were not allowed to evolve in their virulence when they had to attack infected hosts, although this has been observed in real situations (Dion et al. 2011; Rouchet and Vorburger 2014). Table 1 lists all parameters of the simulation model, with their meaning and the values used and Figure 1 gives the flowchart of the different events in the model.

**TABLE 1:** Definition of all parameters of the model with the values used.

Parameter	Meaning	Values used
Hosts	Number of hosts in all simulations and all generations	200
Parasitoids	Number of parasitoid females in all simulations and all generations	10
p	Proportion of hosts infected in the population	Initial level = 0.20
a	Infected host spatial aggregation level, corresponding to a percentage of the number of infected hosts used to compute the number of infected host patches.	0.5% (highly aggregative) 4% (aggregative) 100% (random)
$S_{\mathrm{un}}$	Average of the Exponential distribution used to draw randomly the longevity of uninfected hosts (in number of time steps).	150
$\lambda_{\mathrm{un}}$	Average of the Poisson distribution used to draw randomly the fecundity of uninfected hosts (in number of progeny).	60
c	Cost of being infected for the hosts, leading to reduced longevity and fecundity (see text).	$\left\{0.2;0.3;0.4;0.5;0.6\right\}$
h	Intensity of the horizontal transmission of infection from infected to uninfected hosts (arbitrary unit).	$\left\{0.0;0.2;0.4;0.6;0.8;0.9;1.0\right\}$
$S_{p}$	Average of the Exponential distribution used to draw randomly the longevity of parasitoid females (in number of time steps).	0 to 300, by step of 20
$\lambda_{p}$	Average of the Poisson distribution used to draw randomly the fecundity of parasitoid females (in number of progeny).	150
R	Rate of resistance of hosts to parasitoid attacks when hosting symbionts.	0.0 to 1.0, by step of 0.1
μ	Average of the Normal distribution in which the distance travelled by each parasitoid female foraging for hosts is drawn at each time step (in grid cells).	10
σ	Standard deviation of the Normal distribution in which the distance travelled by each parasitoid female foraging for hosts is drawn at each time step (in grid cells).	5

The model was also used to quantify potential effects of the presence of defensive endosymbionts on the efficacy of biological control against insect pests. For this, different strategies can be used. The efficacy of biological control can be quantified after the host population evolved for 100 generations, as explained above. However, this would simply mean leaving the proportion of hosts infected getting to a stable equilibrium. To control simulations more carefully and thus to understand the obtained results more accurately, the following procedure was used instead. For each replicate of the simulation, and for three different proportions of hosts infected in the population (i.e., 0.2, 0.5 or 0.8), the model was first run during one generation only without parasitoid females and the total host progeny produced was counted to serve as a control. Then, the model was re‐run, again for just one generation, but this time with the presence of the parasitoid females. The percent reduction of host progeny produced is then used to estimate the pest control efficacy in all parameter combinations. This procedure is used with different levels of spatial aggregation of infected hosts and with different intensity of horizontal transmission of infection from infected to uninfected hosts.

In all situations tested (see the corresponding parameter values Table 1), 100 replicates are done, and the results are presented in terms of mean ± SE. Finally, several preliminary tests demonstrated that changes in: the size of the grid defining the environment, the number of hosts, the longevity and fecundity of the hosts, the number of parasitoid females, their fecundity, their distance travelled at each time step, and the initial level of endosymbiont infection can have significant quantitative effects on the results obtained, but led to a change in scale only, without qualitatively affecting the results obtained. Variations in these parameters are thus not presented in the work.

## 3 Results

**FIGURE 2:**
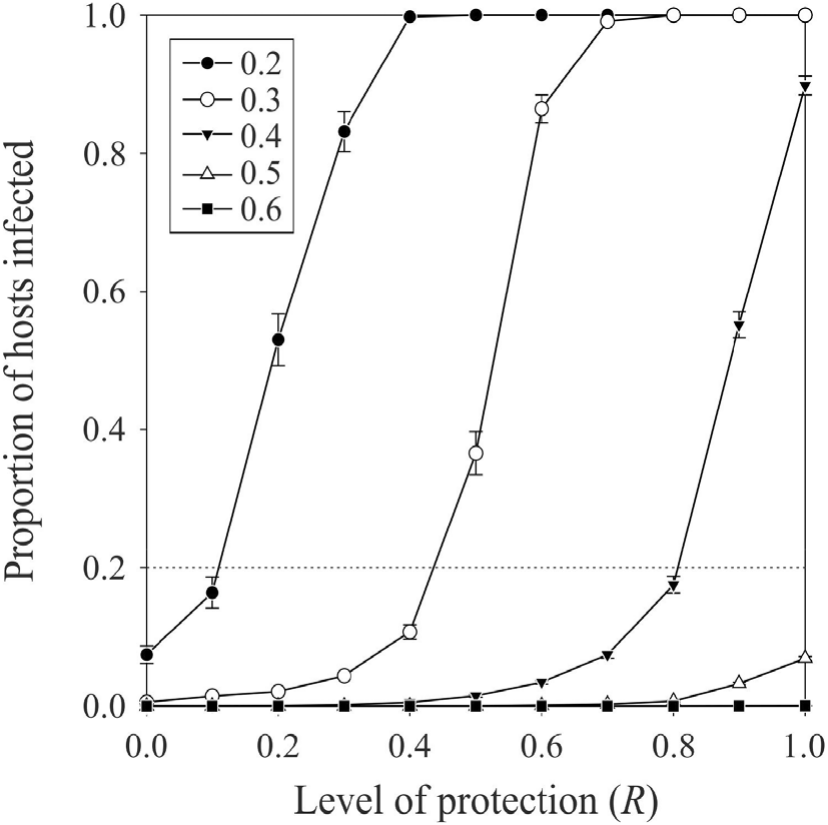
Average (± SE; n=100 in each case) proportion of hosts infected after 100 generations of simulation with different levels of protection of the hosts to parasitoid attacks when hosting symbionts and for different costs of being infected (see legend). Simulations were done with an aggregative level of infected hosts (see Table 1), an intensity of the horizontal transmission of infection from infected to uninfected hosts of 0.8, and an average parasitoid female longevity of 100 (see Table 1 for the value of the other parameters used). The horizontal dotted line corresponds to the initial proportion of hosts infested in the population.

Changing the values of the parameters of the model (see Table 1) led to strong variations in the steady state rate of infected hosts attained after 100 generations of simulation. When the level of protection provided by the symbionts against parasitism is low, being infected or not by the symbionts has (as expected) no consequences for the hosts‐parasitoids dynamics (Figure 2). However, because the hosts are paying a cost for being infected, the rate of infection in the host population declines to low values. When being infected confers resistance to parasitoid attacks, however, infected hosts have a higher reproductive efficiency and the infection level increases up to fixation in the host population. As would be expected, this process is stronger when the cost of being infected is lower.

**FIGURE 3:**
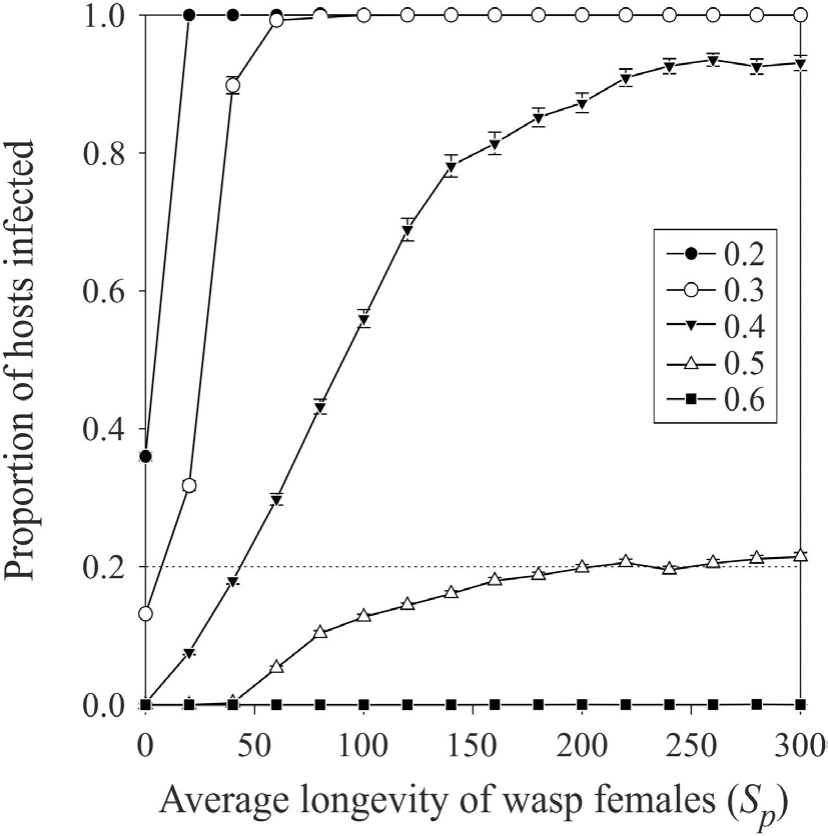
Average (± SE; n=100 in each case) proportion of hosts infected after 100 generations of simulation with different average longevity of the parasitoid females and for different costs of being infected (see legend). Simulations were done with an aggregative level of infected hosts (see Table 1), an intensity of the horizontal transmission of infection from infected to uninfected hosts of 0.8, and a rate of resistance of hosts to parasitoid attacks when hosting symbionts of 0.9 (see Table 1 for the value of the other parameters used). The horizontal dotted line corresponds to the initial proportion of hosts infested in the population.

When the parasitoid females had a low average longevity, which corresponds to a short‐lasting parasitoid pressure on the hosts during simulations, most of the hosts, infected or not, remained unattacked (Figure 3). Here too, since the hosts are paying a cost for hosting symbionts, the level of infection in the host population remains low. When the average longevity of the females increases, however, leading to an increase in the parasitoid pressure, infected hosts are producing on average more progeny than uninfected ones, leading the infection level to increase up to fixation. This effect is again more pronounced with low costs paid by the hosts when they are infected.

**FIGURE 4:**
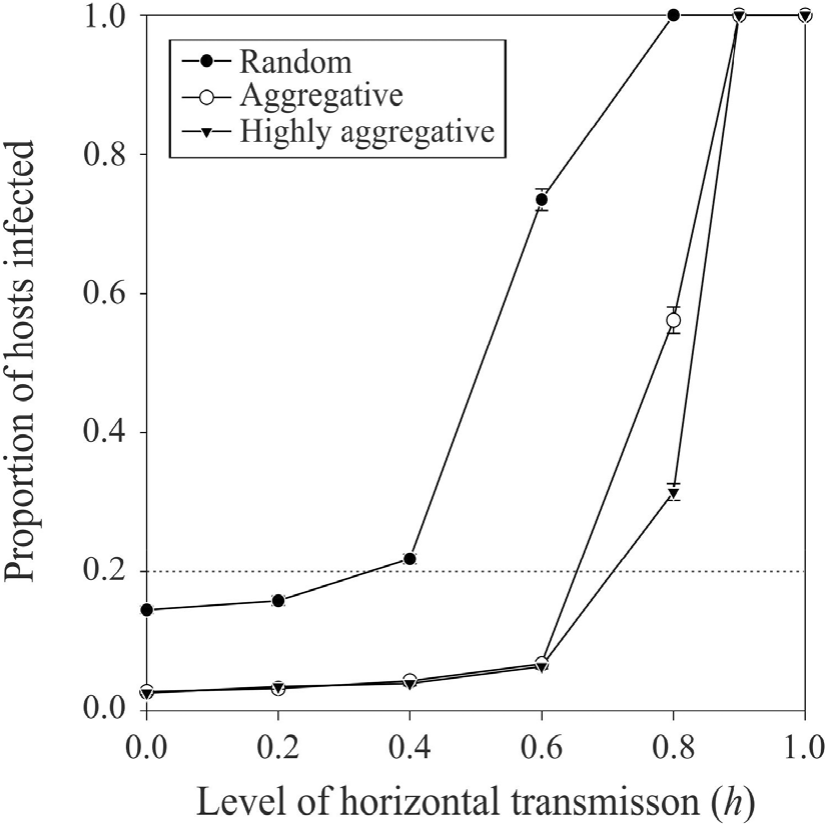
Average (± SE; n=100 in each case) proportion of hosts infected after 100 generations of simulation with different intensity of horizontal transmission of infection from infected to uninfected hosts (arbitrary unit) and for different infected host spatial aggregation levels (see legend). Simulations were done with a cost of being infected of 0.4, and an average female parasitoid longevity of 100 (see Table 1 for the value of the other parameters used). The horizontal dotted line corresponds to the initial proportion of hosts infested in the population.

Increasing the intensity of symbiont horizontal transmission leads to higher rates of symbiont infection in the host population (Figure 4). However, this effect is considerably limited when the infected hosts have an aggregated spatial distribution in the environment. When hosts are clumped, higher levels of symbiont horizontal transmission lead to spread the infection only within host patches, but the overall number of hosts infected in the entire host population remains relatively limited.

**FIGURE 5:**
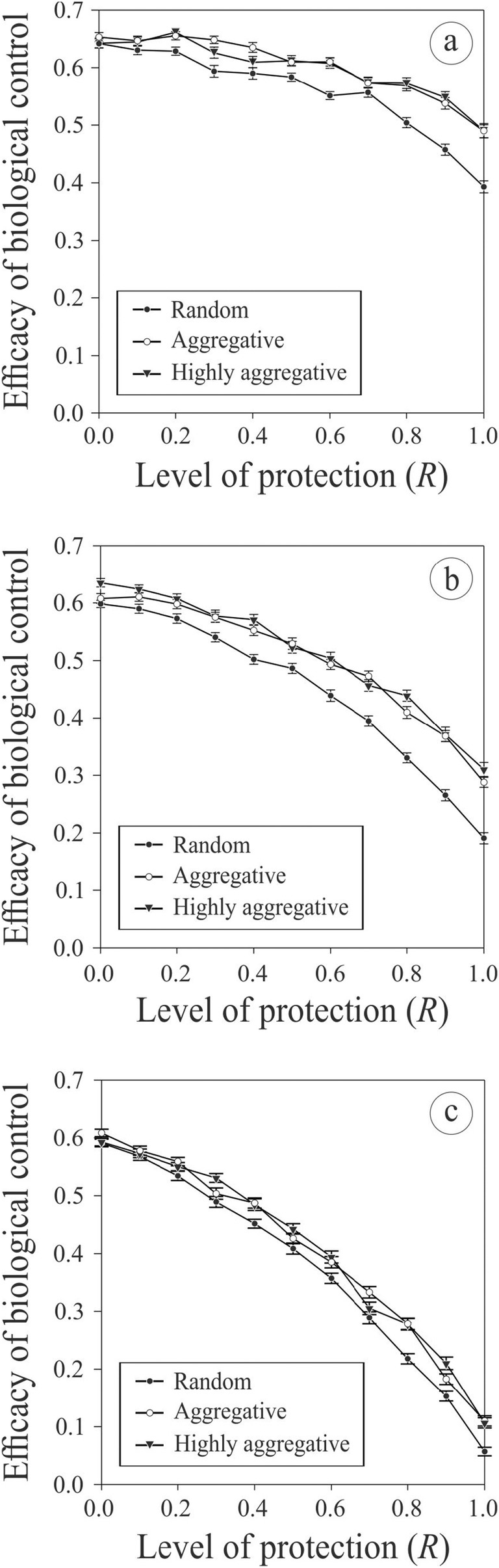
Average potential pest control efficiency (± SE; n=100 in each case) with different levels of protection of the hosts to parasitoid attacks when hosting symbionts and for different infected host spatial aggregation levels (see legend). Simulations were done with an initial proportion of infected hosts in the population of 0.2 (a), 0.5 (b) or 0.8 (b), with a cost of being infected of 0.4, an intensity of the horizontal transmission of infection from infected to uninfected hosts of 0.8, and an average longevity of the parasitoid females of 100 (see Table 1 for the value of the other parameters used).

**FIGURE 6:**
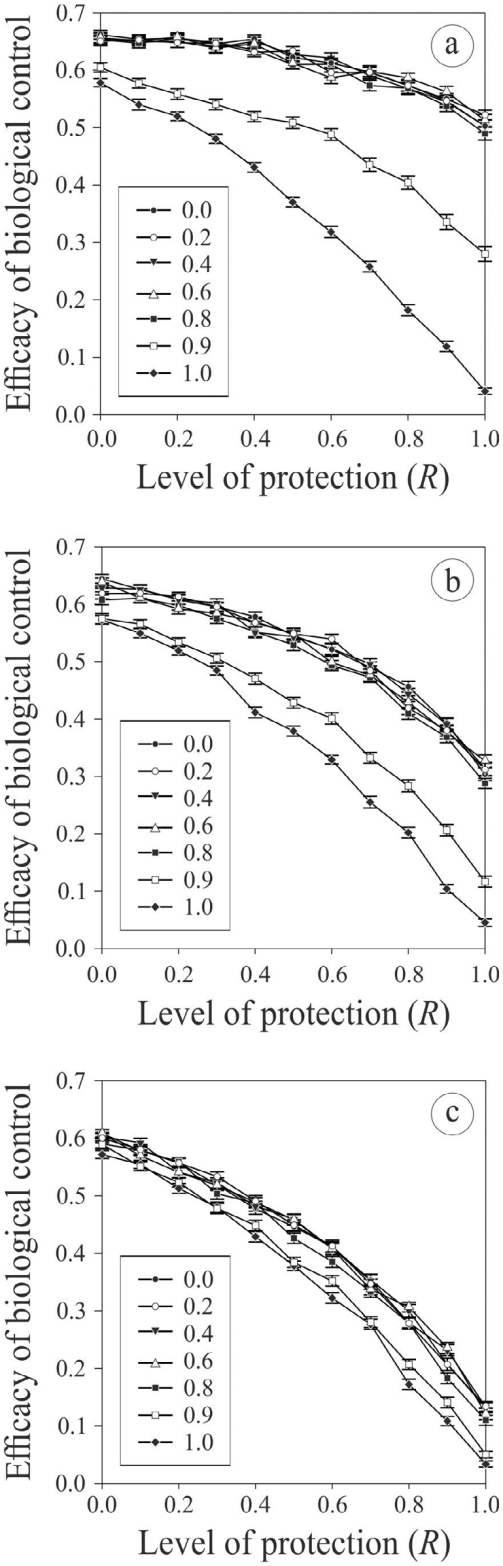
Average potential pest control efficiency (± SE; n=100 in each case) with different levels of protection of the hosts to parasitoid attacks when hosting symbionts and for different intensity of horizontal transmission of infection from infected to uninfected hosts (arbitrary unit; see legend). Simulations were done with an initial proportion of infected hosts in the population of 0.2 (a), 0.5 (b), or 0.8 (c), with an aggregative level of infected hosts (see Table 1), a cost of being infected of 0.4, and an average female parasitoid longevity of 100 (see Table 1 for the value of the other parameters used).

Finally, hosting symbionts can protect hosts against parasitoid attacks and lead to a decline in potential biological control efficacy as the level of protection conferred by the symbionts increases (see Figures 5 and 6). However, this effect is obviously less pronounced if the initial proportion of infected hosts in the population is low, and is also less pronounced if the infected hosts have clumpy spatial distribution patterns (Figure 5), which limits the spread of the infection (see above), and if the intensity of the horizontal transmission from uninfected to infected host is weak (Figure 6). If the level of horizontal transmission is high, then pest control efficacy drastically declines. However, in all other cases, the efficacy of biological control is not strongly affected.

## 4 Discussion

As expected, variation in the protection conferred by symbionts to their hosts against parasitoid attacks had a strong and direct effect on the steady equilibrium of the proportion of hosts infected attained after several generations in our simulations. However, the average longevity of the parasitoid females, a life history trait that has not previously been considered in this context, clearly also needs to be considered. Parasitoid longevity was a parameter in the model developed by Kwiatkowski and Vorburger (2012) but variations in this trait were not studied. Using population cage experiments, Oliver et al. (2008) observed a dramatic increase in the infection level of *Acyrthosiphon pisum* by *Hamiltonella defensa* after repeated exposures to the parasitoid *Aphidius ervi*. In the experimental protocol used by these authors, parasitism was done by allowing female wasps to attack aphids for 24 h on six occasions over about 12 weeks. We assume that all female wasps remained alive during all bouts of parasitism, and this might correspond to a long wasp females' longevity. According to the results presented here, using short‐lived females would have led to a slower increase in endosymbiont infection levels. In all cases, when a parasitoid species or strain has to be chosen to be released for a biological control programme against pests hosting symbionts that can protect them, the results presented here seem to indicate that it would be preferable to use females with intermediate‐level longevity to avoid fixation of symbiont infection in the population, while keeping an efficient pest control efficacy.

Our simulations also indicate that both the level of horizontal transmission of symbionts from infected to uninfected hosts and the host spatial aggregation pattern have strong effect on the dynamics of symbiotic infection. Although the importance of horizontal transmission was discussed on several occasions (Moran and Dunbar 2006; Oliver and Higashi 2019; Brown et al. 2020; Kaech and Vorburger 2021; Kwiatkowski and Vorburger 2012), the consequences of the spatial distribution of hosts, and especially their aggregation pattern, was never formally addressed. Aphids are distributed aggregately in the environment (see, e.g., Nilsen et al. 2013), and such a clumpy distribution is shown here to reduce the overall host infection level since potential horizontal transfers will have an overall lower chance to occur. Also, levels of horizontal transfer are usually estimated to remain moderated (Oliver et al. 2008; Kwiatkowski and Vorburger 2012). Even if hosts can become infected via horizontal transmission, symbionts must successfully establish within a new host, which may occur infrequently (Brown et al. 2020). Finally, even if a symbiont can establish in a host, it is not guaranteed that its protective function against parasitoid attacks is maintained (Brown et al. 2020). Considering all these arguments into account, it is most likely that horizontal transmission would actually play a negligible role on the level of host infection in aphid populations.

Explaining the high amount of observed variation in levels of endosymbiont infection in insect populations is challenging because they are transmitted vertically with high fidelity and they are conferring resistance to parasitoid attacks. Even if the protection is not always perfect, symbiont infection levels should still rapidly spread to fixation (Kwiatkowski and Vorburger 2012). However, intermediate levels of infection are regularly observed in natural populations (Tsuchida et al. 2002; Simon et al. 2003; Vorburger et al. 2009; Oliver and Higashi 2019). The usual explanations for this are that (1) the vertical, maternal symbionts transmission might actually not be perfect; (2) there is a cost for the hosts to harbour symbionts and/or (3) symbionts might potentially be lost (Kwiatkowski and Vorburger 2012). It is possible that coexistence of uninfected and infected hosts could be achieved when there is a very fine equilibrium between costs of infection and the protection level conferred by the symbionts against parasitoid attacks (Kwiatkowski and Vorburger 2012). The results presented here seems to suggest that some other effects might be involved, like variation in life history parameters, especially the longevity, of the parasitoid females, and the spatial distribution of the hosts. In fact, in all of our simulations, a large range of values for the model's parameters led to situations of stable intermediate levels of infection.

Because symbionts can confer a resistance to their hosts against parasitoid attacks, there have been concerns that this might thwart the efficacy of biological control programmes against crop pests (Vorburger 2014, 2018; Oliver and Higashi 2019; Zytynska and Meyer 2019). Several strategies have been proposed to avoid such a problem, but no accurate formal quantification was proposed. According to our results, the main situation in which the defensive endosymbionts can hamper the efficacy of biological control programmes is when they are often transmitted horizontally from infected to uninfected hosts. However, as discussed above, the effect of horizontal transmission is likely negligible, especially when pests are aggregated in the environment. Hence, out results suggest that a large loss in biological control efficacy due to defensive endosymbionts might actually be unlikely. If we also consider the fact that parasitoids are known to evolve countermeasures against the protective effects of endosymbionts (Dion et al. 2011; Rouchet and Vorburger 2014; Vorburger and Perlman 2018; Rossbacher and Vorburger 2020), then the reduction in the efficacy of biological control programmes may actually be even lower. All of this might explain why the occurrence of protective endosymbionts has not been clearly shown to jeopardise the efficacy of biological control programmes under field conditions (Vorburger 2018).

To keep the model tractable and especially sufficiently general, we have made several simplifying assumptions. For example, the values of the model parameters used are expressed in units that do not necessarily correspond to real situations. The goal was not necessarily to carefully describe a real biological system, but to examine whether there were possible situations in which stable intermediate infection levels could be obtained, and/or whether qualitative predictions could be drawn regarding the efficacy of biological control programmes against insects hosting defensive symbionts. The results presented here suggest that this is indeed the case, and both the longevity of the parasitoid females and an aggregated spatial distribution of hosts should be taken into account, especially regarding pest control efficacy, although, as stated above, the level of host aggregation should likely play a minor role in the steady host infection level equilibrium achieved. However, it is possible that important ingredients are missing and should be considered in future developments. For example, host insects in our model are mostly sessile, while there are host‐parasitoid systems in which hosts are highly mobile. Considering host movement could reduce their level of aggregation, and the results would more closely approach what we obtained when the hosts are arranged in a random spatial distribution. Also, in real situations, and as discussed above, parasitoids can evolve to maintain virulence against hosts harbouring defensive endosymbionts (Dion et al. 2011, Rouchet and Vorburger 2014, Vorburger and Perlman 2018, Rossbacher and Vorburger 2020). Taking the evolution of parasitoid virulence into account in the model will most likely lead to a reduction in the steady state infection levels obtained. Despite these missing components, the model presented in this work likely captures some of the important mechanisms involved in the dynamics of insects and their facultative defensive endosymbiont bacteria. We call for verification of this model's predictions in laboratory and field experiments.

## Author Contributions

**Eric Wajnberg:** conceptualization (lead), formal analysis (lead), investigation (equal), methodology (lead), software (lead), supervision (equal), validation (equal), visualization (equal), writing – original draft (equal), writing – review and editing (equal). **Fernando L. Cônsoli:** conceptualization (supporting), formal analysis (supporting), investigation (equal), methodology (supporting), supervision (equal), validation (equal), visualization (equal), writing – original draft (equal), writing – review and editing (equal).

## Conflicts of Interest

The authors declare no conflicts of interest.

## Supporting information


Data S1.

## Data Availability

The code of the simulation model is available from the Dryad Digital Repository: https://datadryad.org/stash/share/‐brhjBaaBrdHH1DniJ0BuaUqSAG8znl7N4SPhVgkBMA.
